# Effect of RNA splicing machinery gene mutations on prognosis of patients with MDS: A meta-analysis

**DOI:** 10.1097/MD.0000000000015743

**Published:** 2019-05-24

**Authors:** Xiaoxue Wang, Xiaomeng Song, Xiaojing Yan

**Affiliations:** Department of Hematology, The First Affiliated Hospital of China Medical University, Shenyang, Liaoning, China.

**Keywords:** MDS, meta-analysis, RNA splicing machinery, SF3B1, SRSF2, U2AF1, ZRSR2

## Abstract

**Background:**

Gene mutations with important prognostic role have been identified in patients with myelodysplastic syndrome (MDS). We performed a meta-analysis to investigate the effects of RNA splicing machinery gene mutations on prognosis of MDS patients.

**Methods:**

We searched English database including PubMed, Embase, Cochrane Library for literatures published within recent 10 years on the effect of RNA splicing machinery genes in MDS. Revman version 5.2 software was used for all the statistical processing. We calculated risk ratio and 95% confidence interval (CI) of continuous variables, and find hazard ratio (HR) and 95% CI of time-to-event data.

**Results:**

We included 19 studies enrolling 4320 patients. There is a significant superior overall survival (OS) in splicing factor 3b, subunit 1 (SF3B1)-mutation group compared to unmutated group (HR = 0.58, 95% CI: 0.5–0.67, *P* < .00001); OS decreased significantly in serine/arginine-rich splicing factor 2/ U2 auxiliary factor protein 1 (SRSF2/U2AF1) mutation group compared to unmutated group, (HR = 1.62, 95% CI: 1.34–1.97, *P* < .00001 and HR = 1.61, 95% CI: 1.35–1.9, *P* < .00001, respectively). In terms of leukemia-free survival (LFS), the group with SF3B1 mutation had better outcome than unmutated group, HR = 0.63 (95% CI: 0.53–0.75, *P* < .00001). Other RNA splicing gene mutation group showed significant poor LFS than unmutated groups, (HR = 1.89, 95% CI: 1.6–2.23, *P* < .00001; HR = 2.77, 95% CI: 2.24–3.44, *P* < .00001; HR = 1.48, 95% CI: 1.08–2.03, *P* < .00001; for SRSF2, U2AF1, and zinc finger CCCH-type, RNA binding motif and serine/arginine rich 2 [ZRSR2], respectively). As for subgroup of low- or intermediate-1-IPSS risk MDS, SRSF2, and U2AF1 mutations were related to poor OS. (HR = 1.83, 95% CI: 1.43–2.35, *P* < .00001; HR = 2.11, 95% CI: 1.59–2.79, *P* < .00001 for SRSF2 and U2AF1, respectively). SRSF2 and U2AF1 mutations were strongly associated with male patients. SF3B1 mutation was strongly associated with disease staging.

**Conclusion:**

This meta-analysis indicates a positive effect of SF3B1 and an adverse prognostic effect of SRSF2, U2AF1, and ZRSR2 mutations in patients with MDS. Mutations of RNA splicing genes have important effects on the prognosis of MDS.

This work was supported by the National Youth Top-notch Talent of Ten Thousand Talent Program and the National Natural Science Foundation of China (NSFC, 81170519).

## 1 Introduction

Myelodysplastic syndrome (MDS) is a kind of myeloid neoplasms characterized by ineffective hematopoiesis, morphologic dysplasia, and cytopenias, which has a high risk of progression to acute myeloid leukemia (AML). The risk stratification for MDS patients is categorized according to clinical characteristics of peripheral blood and bone marrow, also the karyotypes.^[1]^

In recent years, with the development of next-generation sequencing, epigenetic abnormalities and gene mutations in MDS have been gradually summarized. 80% to 90% of patients show at least 1 mutation in one of the >100 addressed genes, supporting the clonal hematopoiesis of the disease and with the diagnosis. Furthermore, it has been demonstrated that the increasing number of gene mutations correlates with the disease outcome in MDS patients.^[2,3]^

RNA splicing machinery plays an important role in the maturation procedure of messenger RNAs (mRNAs). More than 90% of human genes could be affected by the splicing process which may lead to gene expression diversity.^[4]^ Recurrent somatic mutations including splicing factor 3b, subunit 1 (SF3B1), serine/arginine-rich splicing factor 2 (SRSF2), U2 auxiliary factor protein 1 (U2AF1), and zinc finger CCCH-type, RNA binding motif and serine/arginine rich 2 (ZRSR2) which are involved in the RNA splicing machinery have been identified in a considerable number of patients with MDS.^[5]^

Some studies have shown that DNMT3A, TET2, AXSL1, and other gene mutations are associated with the prognosis of MDS.^[6–8]^ However, there is still a lack of systematic studies on RNA splicing gene mutations and clinical relevance. We summarize relevant studies in recent years and summarize the effects of such mutations on the overall survival (OS), leukemia-free survival (LFS), and other clinical characters in order to provide new insight for the diagnosis, treatment, and prognosis of MDS.

## 2 Methods

### 2.1 Retrieval strategy

We searched English database including PubMed, Embase, Cochrane Library for literatures published within recent 10 years on the effect of RNA splicing machinery genes in MDS, using the search strategy “(SF3B1 OR SRSF2 OR U2AF1 OR ZRSR2) AND (MDS OR Myelodysplastic syndrome).” Through the reading of titles and abstracts, the documents are screened and the full texts are read. The appropriate documents are selected according to the inclusion and exclusion criteria. We also searched relevant literature from references available to prevent the omission of the literature. For the raw data not provided in the literature, strive to contact the author for the access.

### 2.2 Literature inclusion criteria and exclusion criteria

Inclusion criteria:

(1) study requires the use of second-generation sequencing to detect prognostic gene mutations. Study must be focused on at least one of the splicing machinery genes mutation (SF3B1, U2AF1, SRSF2, or ZRSR2).
(2) research objects: according to the WHO classification, confirmed the diagnosis of MDS patients;
(3) the article must be published in the form of English;
(4) the study must include at least 1 of the following index as therapeutic evaluation data: OS, transformation time to leukemia (TTL), LFS, and CR.

Reported data could be used to calculate the hazard ratio (HR) with 95% confidence intervals (CIs).

The exclusion criteria:

(1) the expert review, case summary, case report, meeting records;
(2) studies with insufficient data for calculation of incidence and/or HR with 95% CIs; (3) the results of the study does not include any effect of splicing machinery genes mutations on OS, TTL, or LFS.

If more than one published article is from the same study, the results of the most recently published studies should be considered; if the recent articles do not provide definite results, the results of the previous articles are used.

### 2.3 Literature effect index

The clinical effect of different regimens were evaluated by the following indexes: the main effect indicators

(1) OS,
(2) LFS.

Secondary effect indicators PFS/EFS/DFS, CR.

### 2.4 Data extraction

According to the retrieval strategy and retrieval database, 2 researchers independently searched and excluded the literature which did not meet the inclusion criteria. The data extracted from the literature included: author, publication time, regions, ages, sex, classifications, stratifications, average follow-up time, numbers of CR, and other indicators. The results of multivariate analysis were preferred.

### 2.5 Quality assessment and control

All the titles and abstracts of retrieved articles were independently reviewed by 2 investigators (WXX and YXJ) for the inclusion/exclusion criteria. Any divergent opinions were resolved through discussion. The Newcastle–Ottawa quality assessment (NOS)^[9]^ was used to evaluate the quality of each individual study. The evaluation system has 9 items in total. The total score should be 9 if all the standard has been met. In general, studies with 7 or more scores are considered as high quality.

### 2.6 Statistical analysis

In this study, Revman version 5.2 software was used for all the statistical processing. The heterogeneity between subgroups was evaluated by standard chi-square test and *I*^2^-statistic. When *I*^2^ < 50%, suggests that there is no heterogeneity, using fixed effect model, when *I*^2^ > 50%, indicating the existence of heterogeneity and using random effect model, and identify the source of heterogeneity as far as possible. Based on the research included in the analysis, we calculated risk ratio and 95% CI of continuous variables, and find HR and 95% CI of time-to-event data. If HR cannot be obtained directly from the article, we used the Engauge Digitizer V4.1 calculation method.^[10]^ Funnel plot was used to estimate publication bias. *P* < .05 is statistically significant.

### 2.7 Ethics statement

All data sources and statistical analyses were based on previous published studies; thus, no ethical approval and patient consent were required.

## 3 Results of meta-analysis

### 3.1 The basic situation of literature included

**Figure 1:**
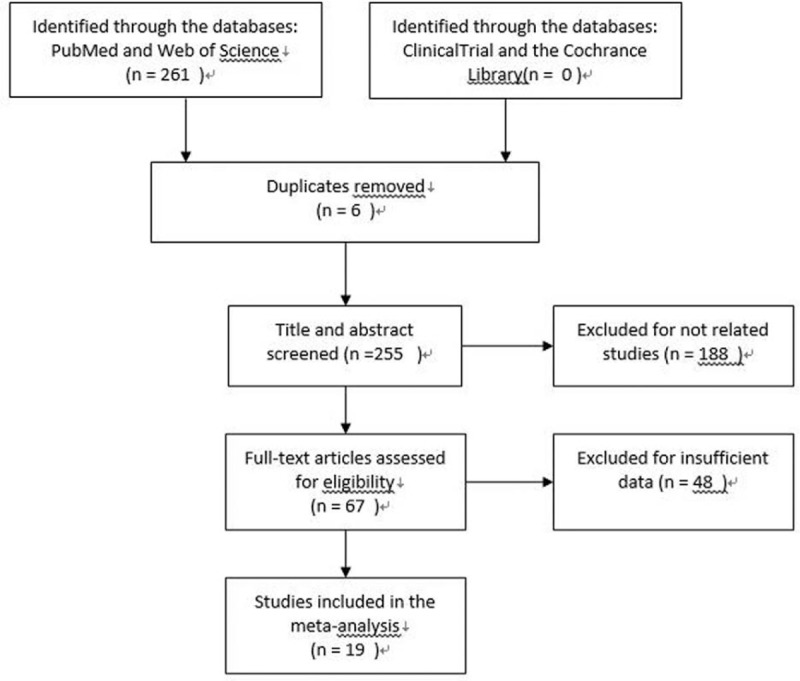
Literature screening flow chart.

A total of 261 articles were retrieved. One hundred eighty-nine articles were excluded by reading titles, abstracts, and types of study. According to inclusion and exclusion criteria, 53 articles were excluded because they did not provide enough information. Finally, 19 articles met the inclusion criteria (Fig. 1).

### 3.2 Characteristics of the studies

There were 11 studies for SF3B1, 9 studies for SRSF2, 9 studies for U2AF1, 2 studies for ZRSR2. A total income of 4320 patients, there were 711 SF3B1 mutations (23.8%), 285 SRSF2 mutations (12%), 231 U2AF1 mutations (8.9%), 31 ZRSR2 mutations (11.1%), and 3062 patients without mutations. The specific characters of the studies can be found in Table 1. NOS was used to evaluate the quality of each study included. The NOS score of each study is shown in Table 2.

**Table 1:**
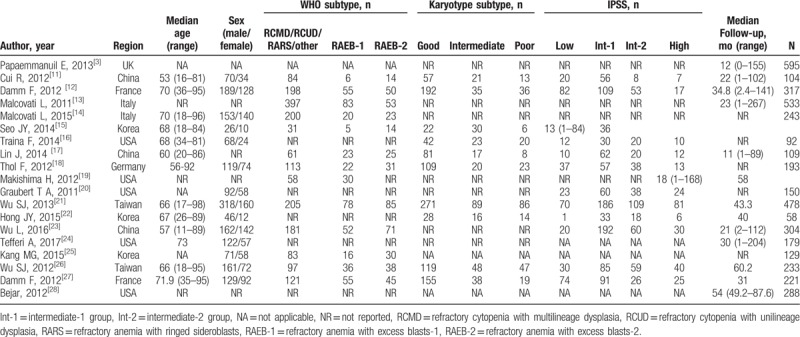
Characteristics of included studies.

**Table 2:**
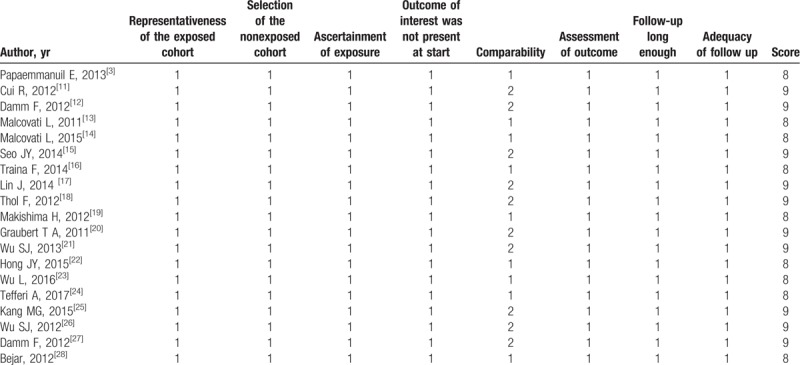
Quality assessment of individual study (NOS, Newcastle–Ottawa quality assessment score.).

### 3.3 Effect index

#### 3.3.1 OS

**Figure 2:**
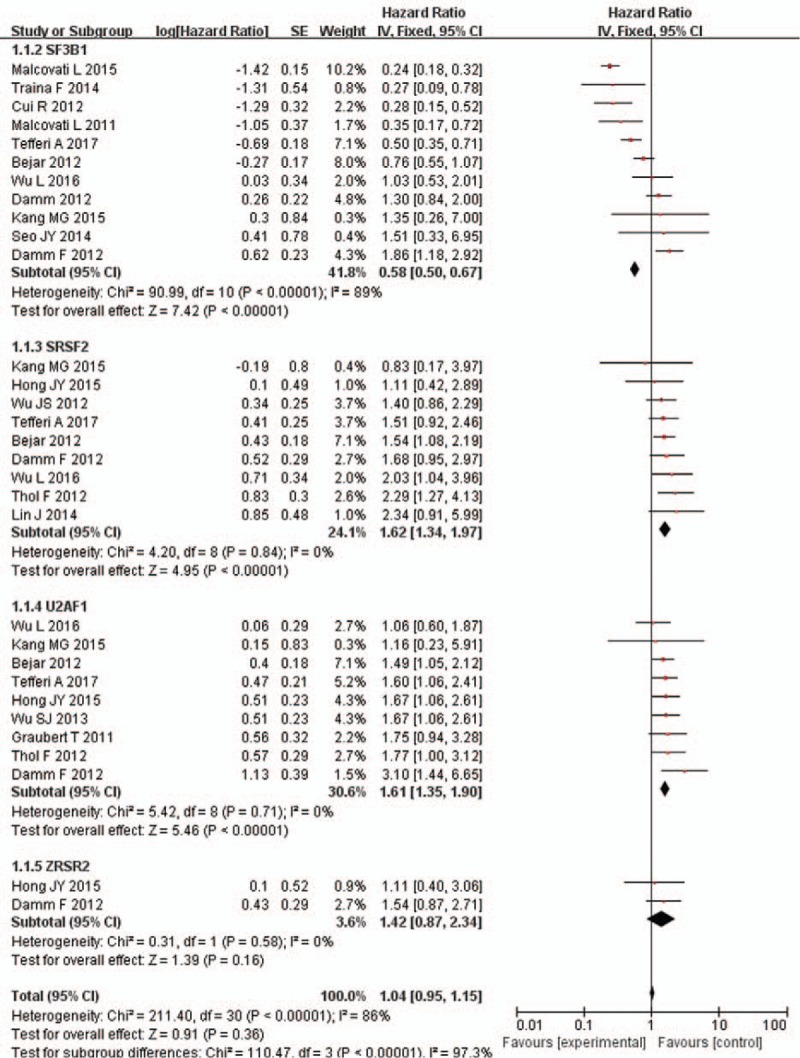
Forest plot of meta-analysis (OS). OS = overall survival.

Nineteen studies all analyzed OS. The meta-analysis performed 11 studies for SF3B1 mutations; 9 studies for SRSF2 mutations; 9 studies for U2AF1 mutations; 2 studies for ZRSR2 mutations. The result of our study showed that patients with SF3B1 mutations could have a better prognosis as regard to OS (HR = 0.58, 95% CI: 0.5–0.67, *P* < .00001), while the heterogeneity is relatively high (*I*^2^ = 89%). On the other hand, an adverse prognostic effect of OS can be observed in the presence of SRSF2/U2AF1 mutations (HR = 1.62, 95% CI: 1.34–1.97, *P* < .00001; HR = 1.61, 95% CI: 1.35–1.9, *P* < .00001, respectively) with no heterogeneity (*I*^2^ = 0%). As for ZRSR2, there is no significant difference on OS comparing ZRSR2-mutation and ZRSR2-unmutation groups (HR = 1.42, 95% CI: 0.87–2.34, *P* = .16, *I*^2^ = 0%). There is significant difference among each subgroup. (*I*^2^ = 97.3%, *P* < .00001). See Figure 2.

#### 3.3.2 LFS

**Figure 3:**
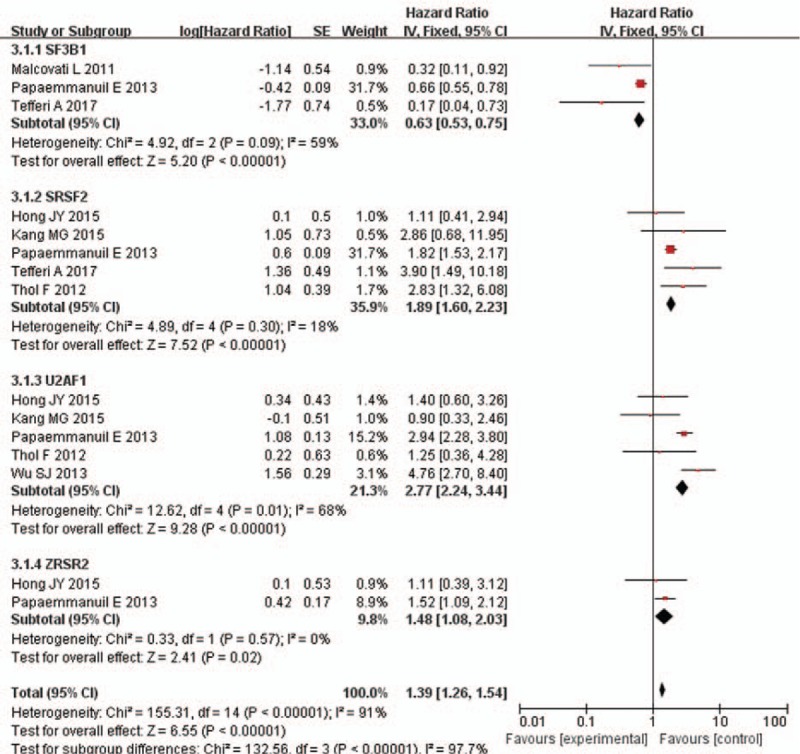
Forest plot of meta-analysis (LFS). LFS = leukemia-free survival.

Seven studies reported data on LFS, with 3 studies focused on SF3B1, 5 for SRSF2, 5 for U2AF1, and 2 for ZRSR2. Our result indicated that patients with SF3B1 mutations were less likely to progress to AML. The pooled HR for LFS is 0.63 (95% CI: 0.53–0.75, *P* < .00001, *I*^2^ = 59%) for patients with SF3B1 mutation compared with unmutated patients. The pooled HR for LFS is 1.89 (95% CI: 1.6–2.23, *P* < .00001, *I*^2^ = 18%) for SRSF2-mutated patients and 2.77 (95% CI: 2.24–3.44, *P* < .00001, *I*^2^ = 68%) for U2AF1-mutated patients and 1.48 (95% CI: 1.08–2.03, *P* < .00001, *I*^2^ = 0%) for ZRSR2-mutated patients, respectively. The results revealed that patients with SRSF2/U2AF1/ZRSR2 mutations were more easily to get transformation to AML compared with unmutated patients. A subgroup analysis showed the presence of severe heterogeneity (*I*^2^ = 97.7%, *P* < .00001). See Figure 3.

### 3.4 OS of low- or intermediate-1 risk MDS

Several studies also summarized the OS data of patients with low/intermediate-1-IPSS risk MDS harboring RNA splicing gene mutations. In this subgroup, patients with SF3B1 mutations did not show any benefit on OS. The pooled HR for OS was 0.96 (95% CI: 0.77–1.2, *P* = .73, *I*^2^ = 67%). As for patients with SRSF2 mutations, the poor prognostic effect can also be observed. The pooled HR for OS was 1.83 (95% CI: 1.43–2.85, *P* < .00001, *I*^2^ = 0%), and the HR for AML transformation was 3.12 (95% CI: 1.37–7.13, *P* = .007, *I*^2^ = 0%) compared with patients without SRSF2 mutations.

**Figure 4:**
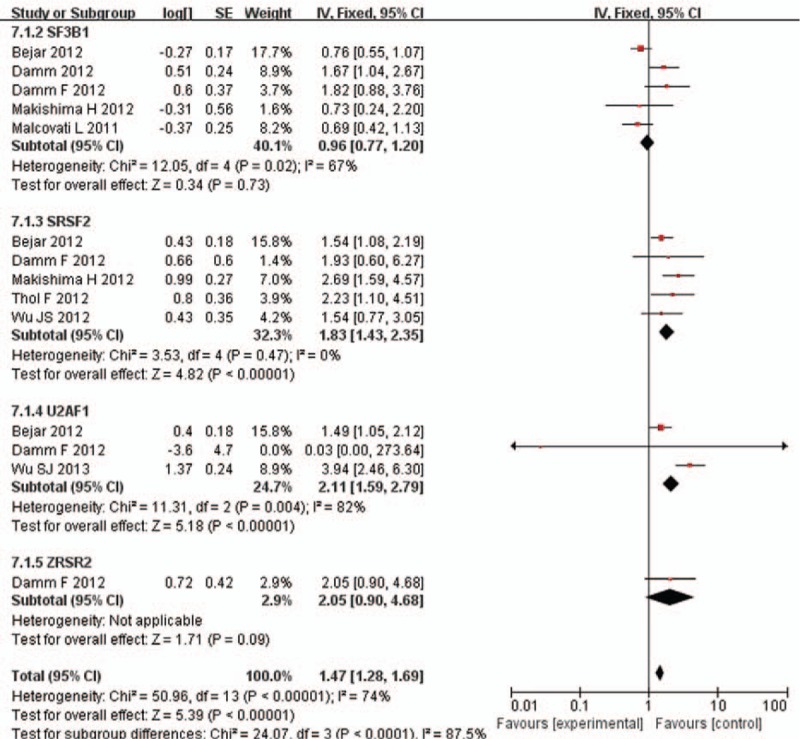
Forest plot of meta-analysis (OS for low-risk MDS). MDS = myelodysplastic syndrome, OS = overall survival.

The results also revealed that patients with U2AF1 mutations had poorer prognosis with regard to OS compared with unmutated group. The pooled HR for OS was 2.11 (95% CI: 1.59–2.79, *P* < .00001, *I*^2^ = 82%). There is only 1 study mentioned effect of ZRSR2 mutation on OS with pooled HR was 2.05 (95% CI: 0.9–4.68, *P* = .09). There is significant difference among each subgroup (*I*^2^ = 87.5%, *P* < .00001) (Fig. 4).

### 3.5 Mutations related to sex and disease staging

**Figure 5:**
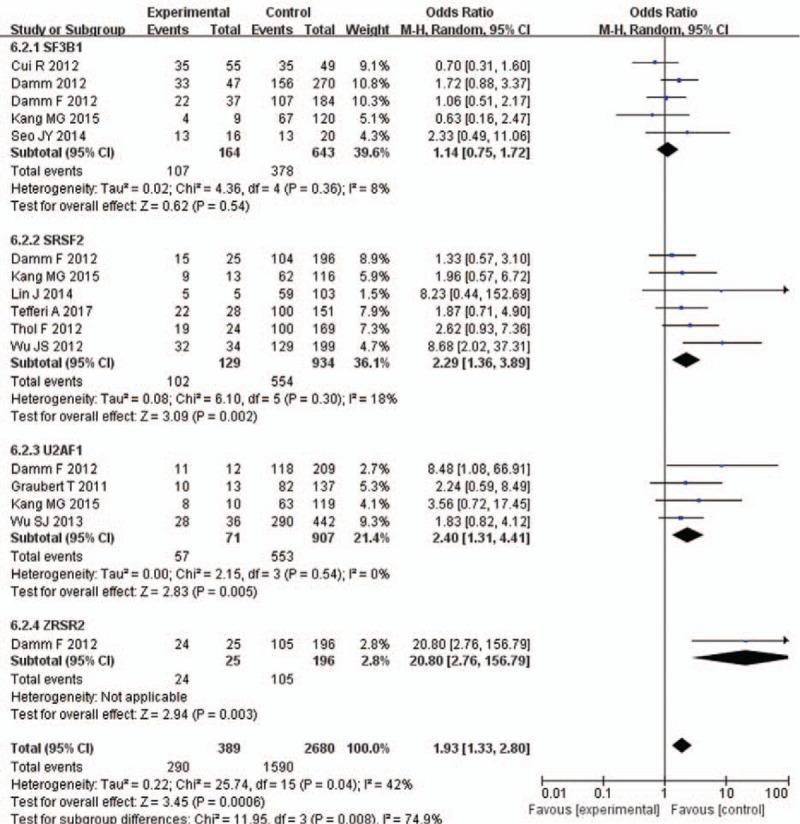
Forest plot of meta-analysis (sex).

SRSF2 and U2AF1 mutations were strongly associated with male sex in some of the included studies. There are more male patients in SRSF2-mutation group than SRSF2-unmutation group (OR = 2.29, 95% CI: 1.36–3.89, *P* = .002), with less heterogeneity (*I*^2^ = 18%). Also, There are more male patients in U2AF1-mutation group than U2AF1-unmutation group (OR = 2.4, 95% CI: 1.31–4.41, *P* = .005), with no heterogeneity (*I*^2^ = 0%). There is no significant difference in sex between SF3B1/ZRSR2 mutation group and SF3B1/ZRSR2 unmutated group (Fig. 5).

**Figure 6:**
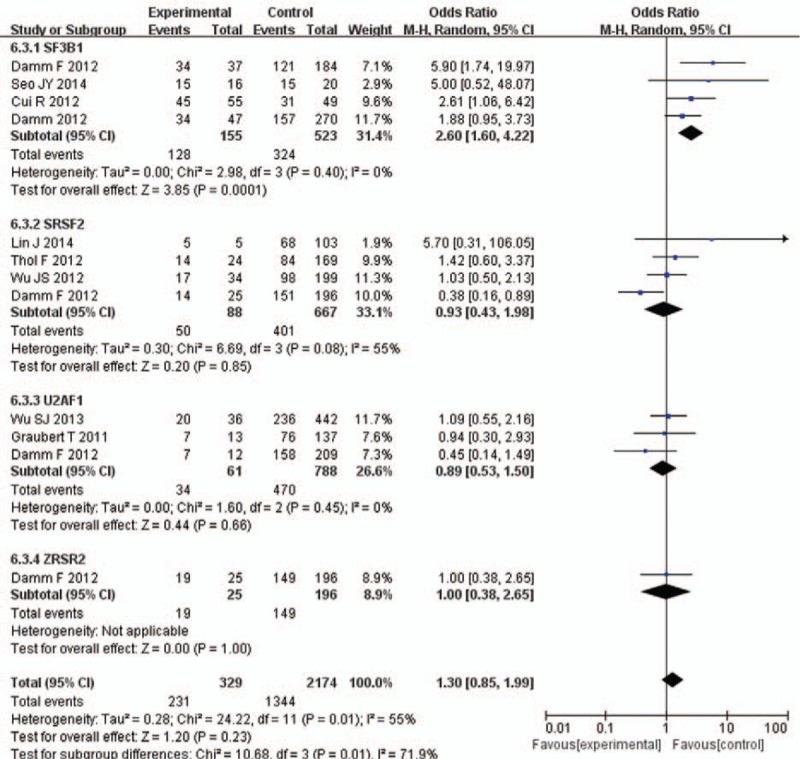
Forest plot of meta-analysis (disease staging).

Four studies indicated SF3B1 mutation was strongly associated with disease staging, but not for other RNA splicing gene mutations (OR = 2.6, 95% CI: 1.6–4.22, *P* = .0001), with no heterogeneity (*I*^2^ = 0%) (Fig. 6).

### 3.6 Publication bias

**Figure 7:**
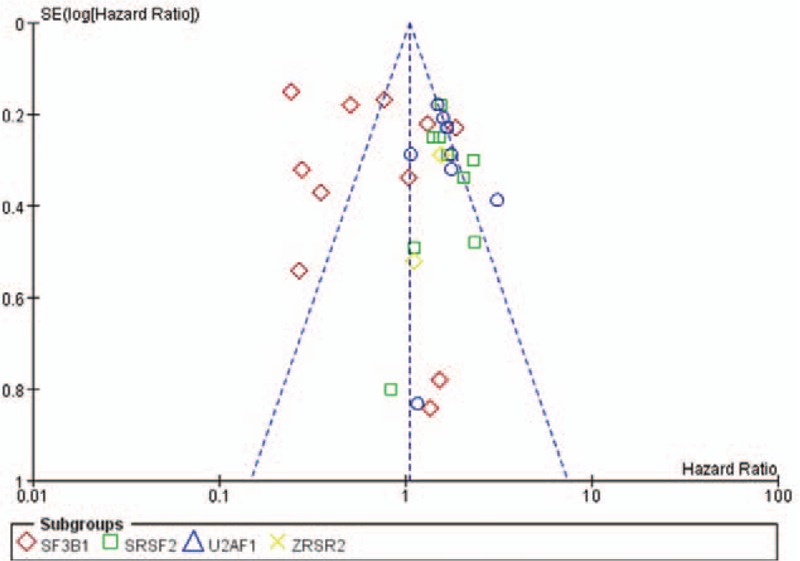
Funnel plot of meta-analysis.

Publication bias could be assessed by funnel plot (Fig. 7). Funnel plots of each analysis did not show significant publication bias in the studies included.

## 4 Discussion

With the development of new drugs (such as hypomethylating agents), the prognosis of MDS has been much better. But not all the patients could benefit from hypomethylating medication. At the same time, allogeneic stem cell transplantation is usually considered for well behaved-patients.^[29]^ Genetic alterations in patients with MDS has been widely concerned because of the significant prognostic effect.^[2]^ Therefore, exploring the prognosis related gene mutations and developing precision therapies based on the risk stratification and potential targets will be of great significance to the overall diagnosis and treatment of MDS.

In the past decade, a series of gene mutations have been identified in MDS, which are involved in different mechanism including signal transduction/kinase (JAK2, KRAS, CBL, etc); DNA methylation (DNMT3A, TET2, IDH1/2); DNA repair like TP53; transcriptional factor regulation (TP53, ETV6, RUNX1, BCOR); cohesion complex (STAG2, CTCF, SMC1A); chromatin modification (EZH2, ASXL1); and RNA-splicing machinery (SF3B1, U2AF1, SRSF2, ZRSR2).^[30]^ Several studies have shown that gene mutations in MDS are closely related to the onset and prognosis of the disease and might be the potential therapeutic targets.^[7,8,31,32]^

The procedure of modification by spliceosomes is of great importance to produce normal mRNAs. Aberrant splicing and mutations have been described in cancer.^[33]^ We may explore new therapeutic targets for patients with MDS through in-depth research on splicing mutations.

SF3B1 is involved in the early stages of spliceosome assembly.^[34]^ Prior studies suggested that SF3B1 mutation can be frequently found in patients with refractory anemia with ring sideroblasts and was likely to have reduced hemoglobin levels. It might be a potential novel marker in the diagnosis of RARS.^[27,35,36]^ SF3B1 mutations were more frequent in –5/5q– cases.^[27]^ A study revealed that patients with SF3B1 mutations had relatively longer event-free survival and fewer cytopenias.^[36]^ Another research showed that the presence of SF3B1 mutations was significantly associated with better overall and leukemia-free survival in RARS and RCMD.^[37]^ Intriguingly, a study showed that SF3B1-mutated patients had a significantly inferior outcome because of an additional aberrant karyotype.^[38]^ SF3B1 mutation can also be detected in patients with MDS-RAEB1/2, but at a relatively low rate. Our study showed similar result that SF3B1 mutation is closely related to the OS and LFS in MDS patients. Furthermore, we found that patients with SF3B1 mutation were strongly associated with disease staging.

SRSF2 encodes serine/arginine-rich splicing factor 2, playing a role in preventing exon skipping and ensuring the accuracy of splicing.^[39]^ SRSF2 mutations occur dominantly in older patients and have higher rates in male population. Several studies revealed that the presence of SRSF2 mutations was sometimes associated with RUNX1, ASXL1, IDH1, and IDH2 mutations.^[18,25,26]^ IDH2 and ASXL1 are generally considered to be associated with poor prognosis in MDS,^[8,40]^ while RUNX1 also showed a poor effect in AML patients.^[41]^ A recent study showed that SRSF2 predicted leukemic transformation and might be an independent factor of prognosis.^[42]^ Another study indicated that most of the patients with SRSF2 mutations belonged to RAEB-1 and RAEB-2 subtypes and had remarkable thrombocytopenia.^[27]^ In our study, SRSF2 mutation showed significant poor prognosis as considering OS and LFS, which also applied for the OS of low-risk MDS. There are more male patients in SRSF2-mutation group than SRSF2-unmutation, the mechanism of which needs further investigation. There is no obvious association between the mutation and disease staging.

U2AF1 is a U2 auxiliary factor protein functions as a recognizer of the AG splice acceptor dinucleotide at the 3′ end of introns and is involved in pre-mRNA processing.^[43]^ Several studies indicated that U2AF1 mutations were associated with younger patients and ASXL1,^[18,27]^ JAK2,^[21]^ or DNMT3A^[18]^ mutations. The JAK-2 V617F mutation which can be easily found in myeloproliferative neoplasm has been reported in a small part of MDS and its prognostic significance is unclear.^[44,45]^ U2AF1 mutations occurred probably more frequently in patients with isolated -20/20q- or trisomy 8 than the others.^[20,21,27]^ It was suggested that U2AF1 mutation was an independent prognostic factor for OS in MDS patients (<50 years).^[21]^ It was also shown that patients with U2AF1 mutations were more likely to progress to AML.^[20]^ In our study, U2AF1 mutations showed significant disadvantage in OS and LFS in MDS patients.

ZRSR2 is involved in splice-site selection, spliceosome assembly, and splicing.^[30]^ Single mutation of ZRSR2 can usually be found in older patients which may lead to macrocytic anemia without dysplasia or other kinds of cytopenia.^[46]^ A study showed a higher rate of AML transformation in a group of patients with ZRSR2 mutation in the IPSS-low/intermediate-1 subgroups.^[27]^ The patients with ZRSR2 mutations exhibited higher blasts in bone marrow, and sometimes neutropenias. We found that ZRSR2 mutation showed significant poor LFS than unmutated groups.

There are few researches focusing on the response to hypomethylating drugs of RNA splicing genes. It has been suggested that U2AF1 mutation was significantly associated with non-response to azacitidine, but for other spicing machinery genes, the results were negative.^[16,47]^ More studies are needed for searching new precision therapeutic strategies for the MDS patients with splicing machinery gene mutations. The limitations of this meta-analysis should be taken into account. Some of the studies contained small amount of patients, thus the result requires confirmation in a larger patient cohort. It also lacked detailed analysis of the association between karyotype abnormalities and prognosis.

## 5 Conclusion

Our study summarized the published literatures and revealed a positive prognostic effect of SF3B1 mutation and an adverse prognostic effect of SRSF2/U2AF1/ZRSR2 mutations in patients with MDS. As for the subgroup of low/intermediate-1-IPSS risk MDS, SRSF2/U2AF1 mutations also indicated poor prognosis. In addition, SRSF2 and U2AF1 mutations were strongly associated with male patients. SF3B1 mutation was strongly associated with disease staging. The mutations of RNA splicing genes may be a promising prognostic factor and therapeutic target to MDS patients. Further clinical trials are needed to better understand the prognostic impact of RNA splicing genes mutations in MDS.

## Author contributions

Xiaoxue Wang and Xiaomeng Song contributed equally to this work.

**Conceptualization:** Xiaojing Yan.

**Data curation:** Xiaomeng Song.

**Formal analysis:** Xiaomeng Song.

**Funding acquisition:** Xiaojing Yan.

**Investigation:** Xiaoxue Wang.

**Resources:** Xiaojing Yan.

**Software:** Xiaomeng Song.

**Supervision:** Xiaojing Yan.

**Validation:** Xiaoxue Wang.

**Visualization:** Xiaoxue Wang.

**Writing – original draft:** Xiaoxue Wang.

**Writing – review and editing:** Xiaojing Yan.
